# DNA Methyltransferase 1 (DNMT1) Shapes Neuronal Activity of Human iPSC-Derived Glutamatergic Cortical Neurons

**DOI:** 10.3390/ijms22042034

**Published:** 2021-02-18

**Authors:** Sarah Bachmann, Jenice Linde, Michael Bell, Marc Spehr, Hans Zempel, Geraldine Zimmer-Bensch

**Affiliations:** 1 Institute of Human Genetics, Faculty of Medicine and University Hospital Cologne, University of Cologne, 50931 Cologne, Germany; sarah.bachmann@uk-koeln.de (S.B.); michael.bell@uk-koeln.de (M.B.); 2 Center for Molecular Medicine Cologne (CMMC), Faculty of Medicine and University Hospital Cologne, University of Cologne, 50931 Cologne, Germany; 3 Functional Epigenetics in the Animal Model, Institute for Biology II, RWTH Aachen University, 52074 Aachen, Germany; jenice.linde@rwth-aachen.de; 4 Research Training Group 2416 MultiSenses-MultiScales, Institute for Biology II, RWTH Aachen University, 52074 Aachen, Germany; M.Spehr@sensorik.rwth-aachen.de; 5 Department of Chemosensation, Institute of Biology II, RWTH Aachen University, 52074 Aachen, Germany

**Keywords:** DNMT1, human iPSC, layer 2/3 cortical neurons, synaptic activity, spontaneous activity, calcium imaging

## Abstract

Epigenetic mechanisms are emerging key players for the regulation of brain function, synaptic activity, and the formation of neuronal engrams in health and disease. As one important epigenetic mechanism of transcriptional control, DNA methylation was reported to distinctively modulate synaptic activity in excitatory and inhibitory cortical neurons in mice. Since DNA methylation signatures are responsive to neuronal activity, DNA methylation seems to contribute to the neuron’s capacity to adapt to and integrate changing activity patterns, being crucial for the plasticity and functionality of neuronal circuits. Since most studies addressing the role of DNA methylation in the regulation of synaptic function were conducted in mice or murine neurons, we here asked whether this functional implication applies to human neurons as well. To this end, we performed calcium imaging in human induced pluripotent stem cell (iPSC)-derived excitatory cortical neurons forming synaptic contacts and neuronal networks in vitro. Treatment with *DNMT1* siRNA that diminishs the expression of the DNA (cytosine-5)-methyltransferase 1 (DNMT1) was conducted to investigate the functional relevance of DNMT1 as one of the main enzymes executing DNA methylations in the context of neuronal activity modulation. We observed a lowered proportion of actively firing neurons upon *DNMT1*-knockdown in these iPSC-derived excitatory neurons, pointing to a correlation of DNMT1-activity and synaptic transmission. Thus, our experiments suggest that DNMT1 decreases synaptic activity of human glutamatergic neurons and underline the relevance of epigenetic regulation of synaptic function also in human excitatory neurons.

## 1. Introduction

All learned behavior and the retrieval of memories, cognition, sensory perception, and motor control depend on the formation of stable synaptic connections to form sustained neuronal ensembles. Even in the adult brain, synaptic contacts show a high degree of plasticity, which resembles a prerequisite for learning and memory formation. While some cellular processes that constitute synaptic plasticity and mediate synaptic functionality, such as postsynaptic receptor activation and the respective downstream signaling cascades, are well investigated, many questions regarding memory formation and the flexibility of synapses remain unresolved [1]. Since neurons are required to develop distinct gene expression profiles to enforce their role in neuronal circuits and memory engrams [2,3], epigenetic mechanisms recently emerged as new examination targets in the regulation of synaptic function and plasticity. Their dynamic nature and responsiveness to altered neuronal activity [4,5,6] could explain the translation of external stimuli, e.g., as presented during learning, into changed gene expression that underlies structural and functional synaptic adaptations.

The toolbox of epigenetic mechanisms comprises, inter alia, DNA methylation, histone modifications, and noncoding RNAs. Their influences on the cellular gene transcription machinery are highly versatile and strongly interconnected, rendering them complex instruments of modulation of gene expression. The methylation of the DNA, occurring mainly at cytosines, is a reversible and dynamic process [4,7,8]. While enzymes of the DNA methyltransferase (DNMT) family catalyze cytosine methylation, active demethylation is executed via oxidation by ten-eleven translocation (TET) proteins and subsequent iterative oxidation and base excision repair [9,10,11]. These mechanisms enable dynamic reconfigurations of DNA methylation signatures, observed during and being proposed to be critical for neuronal differentiation and maturation [12,13,14,15].

In addition to developmental processes, altered DNA methylation signatures were correlated to changes in synaptic transmission [7,16,17] and synaptic scaling [18] of murine hippocampal neurons. As underlying mechanisms, the transcriptional control of plasticity- and synapse-related genes are discussed [19]. We recently showed that DNMT1-dependent DNA methylation modulates the expression of endocytosis-related genes, thereby acting on the replenishment of synaptic vesicles and hence, GABAergic transmission [20].

However, not only does DNA methylation-dependent transcriptional control regulate synaptic functionality and plasticity, synaptic function in turn also influences DNA methylation. The DNA methylation landscape was reported to be remodeled dynamically, e.g., in response to altered neuronal activity in the adult brain [4]. Moreover, the expression of key enzymes required for DNA methylation and demethylation were already shown to be changed in response to neuronal activity [19,21]. For example, activation of synaptic GluN2A-containing N-methyl-D-aspartate receptors (NMDARs) was shown to change DNA methylation signatures by driving proteasomal degradation of the de novo DNA-methyltransferase DNMT3A1 in the adult brain [22].

In summary, activity-dependent modulation of DNA methylation, e.g., by promoting the degradation of DNMTs, represents an appealing hypothesis of how neuronal activity changes the DNA methylation landscape, and through this, acts back on the DNA methylation-dependent regulation of synaptic function and plasticity.

The so far described studies addressing the DNA methylation-dependent regulation of synaptic function were mainly performed in murine neurons. Therefore, one crucial question still remains: to what extent does the acquired knowledge apply to human neurons and the human brain?

In patients suffering from temporal lobe epilepsy (TLE) increased expression of DNMT1 and DNMT3a was observed in the neocortex [23]. Moreover, TLE patients’ seizure rates correlated with changed expression and DNA methylation profiles of endocytosis-related genes shown to be regulated by DNMT1 in murine interneurons [20]. This indicates that similar mechanisms of DNMT-dependent regulation of synaptic function could play a role in human cortical neurons with potential implication in TLE disease pathophysiology

Here, we tested whether *DNMT1* expression levels can indeed influence neuronal activity in human excitatory neurons and found that siRNA-mediated reduction of *DNMT1* expression in iPSC-derived glutamatergic cortical neurons results in decreased spontaneous calcium oscillation. This indicates a positive regulation of neuronal activity by DNMT1, hinting towards a key role of DNMT expression and DNA methylation for the regulation of neuronal activity in human cortical excitatory neurons.

## 2. Results

### 2.1. Generation of Cortical Neurons from Human Induced Pluripotent Stem Cells (iPSCs)

Given the general importance of synaptic activity and plasticity modulation in the mammalian cortex, it seems reasonably probable that the interdependence of DNA methylation and synaptic function and activity is a universal concept in organisms with a plastic nervous system. As the expression of key enzymes required for DNA methylation and demethylation was already shown to be changed in response to neuronal activity [19,21], the role of DNA methylation in integrating information by adapting synaptic functionality dependent on external stimuli could be a general concept across species and neuronal cell types.

**Figure 1:**
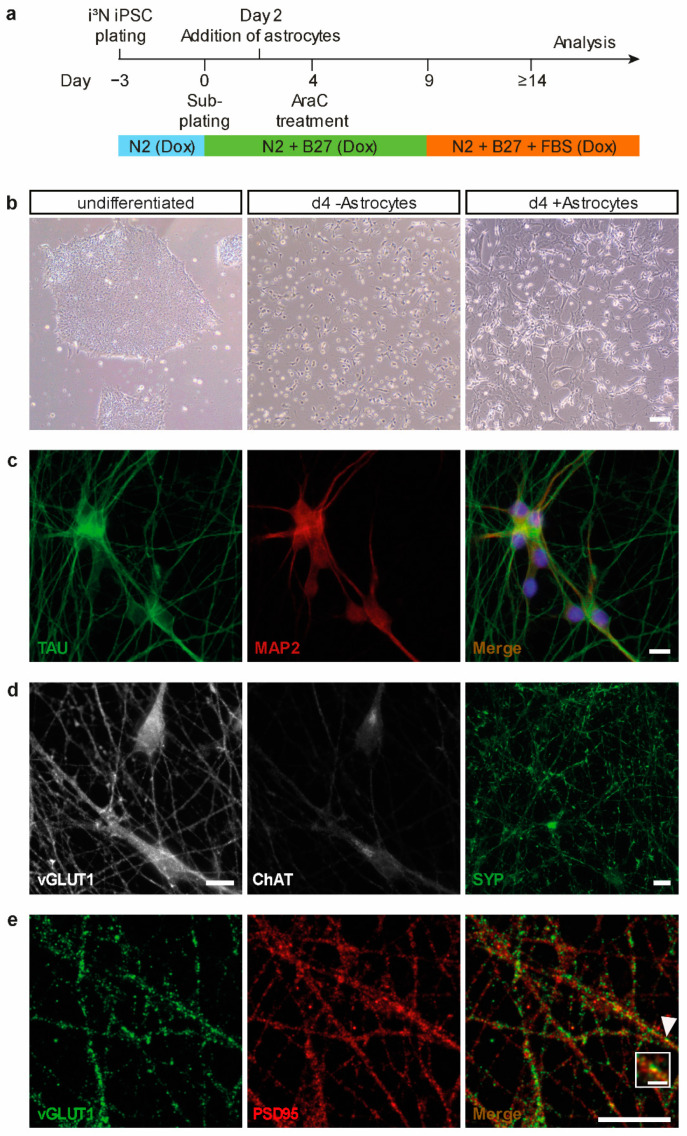
Differentiation and characterization of human induced pluripotent stem cell (iPSC)-derived cortical neurons. (**a**) Schematic representation of human iPSC differentiation protocol using doxycycline and coculturing of primary murine astrocytes. (**b**) Representative phase-contrast images at indicated time points of human iPSC differentiation into cortical neurons with and without coculturing of astrocytes; scale bar = 100 µm. (**c**–**e**) Representative images of iPSC-derived neurons 14 days after initiation of differentiation indicate proper neuronal maturation into glutamatergic neurons, and formation of axons, dendrites and synapses. (**c**) Immunocytochemical stainings of neuronal marker expression (TAU, MAP2) show the expected polarized distribution of predominantly axonal TAU (left panel) and predominantly dendritic MAP2 protein (middle panel). The merge includes nuclear stain NucBlue to indicate somata (right panel); scale bar = 10 µm. (**d**) Immunocytochemical stainings for neuronal differentiation markers. Neurons express glutamate transporter 1 (vGLUT1; left panel) but lack the expression of the motor neuron marker choline acetyltransferase (ChAT; middle panel). Neurons express the presynaptic marker synaptophysin (SYP; right panel); scale bars = 10 µm. (**e**) Immunocytochemical stainings for neuronal synaptic markers. Neurons form synapses, as visualized by expression and co-localization of the signals obtained with antibodies directed against the presynaptic marker vGLUT1 (left panel) and the postsynaptic marker PSD95 (middle panel); white arrowhead in the merge-image (right panel) indicates an example of a mature synapse, which is magnified in the white squared box; scale bar = 10 µm, scale bar of magnification = 1.5 µm.

To approach the functional implication of DNMT1 in activity regulation of human cortical neurons, we made use of human induced pluripotent stem cells (iPSCs) and their potential to differentiate into functional neurons. In general, iPSCs derive from reprogrammed somatic cells, such as fibroblasts, and have the potential to differentiate into all three cell lineages (ectoderm, endoderm, mesoderm). In this study, we used the well-established WTC11 human iPSC line, with an inducible *Ngn2* transgene, which can be easily differentiated into layer 2/3 glutamatergic cortical neurons by the addition of doxycycline [24,25]. Differentiation into cortical neurons was largely performed as previously described [25], but with some modifications (for details, see methods and Figure 1a). After having tested different available neuronal differentiation protocols, we settled for a coculture system with murine astrocytes (Figure 1b) that resulted in a higher percentage of neurons showing reliable neuronal activity as assessed by calcium imaging (Supplementary Files A1 and A2). After two weeks of differentiation, neurons displayed solid expression and proper localization of the neuronal and axodendritic markers TAU and MAP2 (Figure 1c, left, middle panel). We used TAU to visualize the development of axons and the establishment of a complex axonal network [26]. MAP2 immunostaining was conducted to visualize the successful formation of dendrites, as MAP2 is expressed in and localizes to the somatodendritic compartments in mature neurons [27]. A merge of TAU and MAP2 signals, together with nuclear staining using NucBlue to label the somata, confirms the development of in-vivo-like functional neurons (Figure 1c, right panel). As *Ngn2* expression in WTC11 stem cells was reported to yield in highly pure neuronal cultures of glutamatergic neurons [28], the expression of the glutamatergic marker vesicular glutamate transporter 1 (vGLUT1) was further assessed in our neuronal cultures by immunofluorescene (Figure 1d, left panel). Presence of vGLUT1, but the absence of the choline acetyltransferase (ChAT; Figure 1d, middle panel) confirms the expected and previously reported differentiation into glutamatergic neurons [25,28]. Additionally, the expression and staining patterns of the presynaptic markers synaptophysin (Figure 1d, right panel), and vGLUT1 (Figure 1e, left panel) in combination with the expression pattern of the postsynaptic density protein (PSD95) as a postsynaptic marker (Figure 1e, middle panel) indicate the establishment of functional synapses in this human neuronal cell culture model (Figure 1e, right panel).

**Figure 2:**
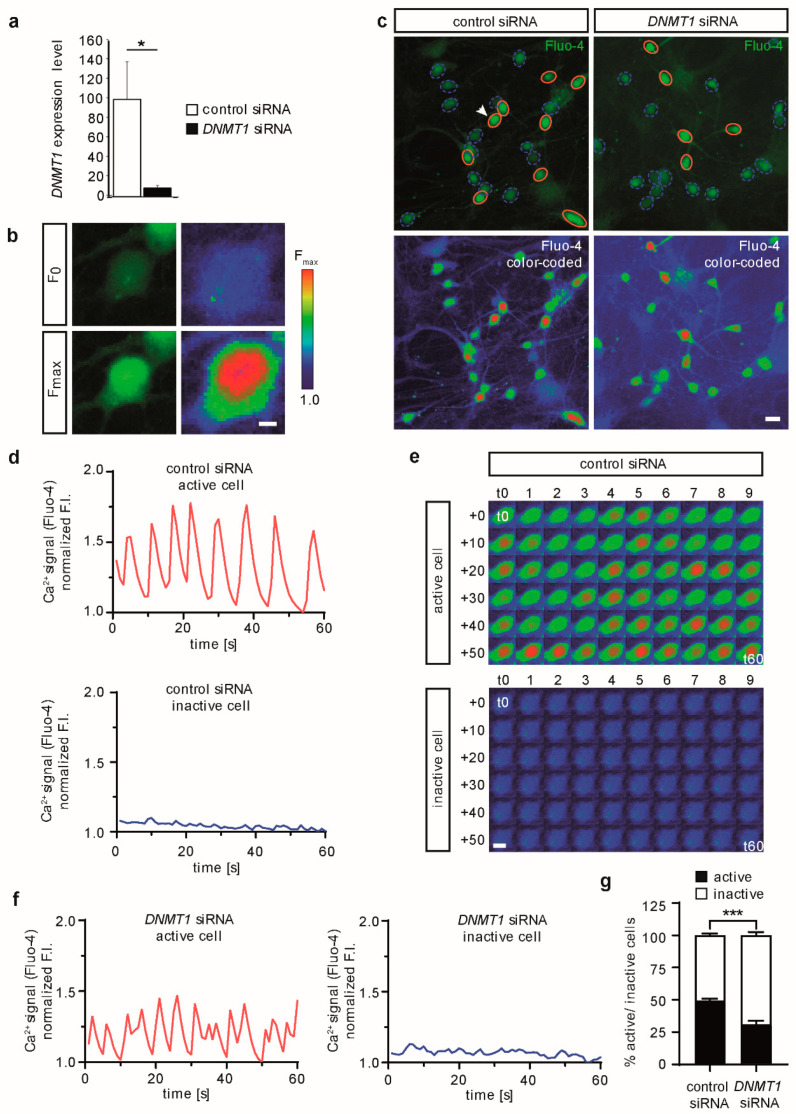
Spontaneous calcium oscillations are reduced in *DNMT1* siRNA-treated human iPSC-derived neurons. Human iPSC-derived neurons (d14/d15) were transfected with control or *DNMT1* siRNA for 24 h. Neurons were loaded with Ca^2+^-sensitive Fluo-4 dye and subsequently used for live-cell imaging of spontaneous calcium oscillations. (**a**) Validation of DNMT1 downregulation via *DNMT1* siRNA. Expression levels of HEK293T cells 24 h after siRNA transfection were assessed by qPCR, using *RPS18* as housekeeping gene for normalization. Bar graphs indicate mean *DNMT1* expression levels ± standard errors; two-tailed Student’s *t*-test, * = *p* < 0.05. (**b**) Representative images of the Fluo-4 fluorescence as recorded during imaging (left images) and visualized via pseudo-coloring (right images) of an oscillating cell in its inactive state (low calcium levels, upper row) and active state (high calcium levels, lower images). F_0_ = minimum fluorescence, F_max_ = maximum fluorescence; scale bar = 5 µm. (**c**) Overview image depicting a single frame of a random example of neurons transfected as indicated (left panels: control siRNA; right panels: *DNMT1* siRNA). Upper panels show fluorescence intensity (FI) of Fluo-4 (green color), active cells are circled in red, inactive cells are circled with blue dashed lines (white arrow points at cell magnified in (**b**)). Lower panels show the same images but with pseudo-colored fluorescence intensity similar as in (**a**); scale bar = 20 µm. (**d**) Representative traces of normalized fluorescence intensity (F.I.) of an exemplary neuron transfected with control siRNA. Upper trace shows an active cell (solid calcium oscillation), lower trace shows an inactive cell (no observable calcium oscillations). (**e**) Serial images of an active (upper panel) and an inactive cell (lower panel) transfected with control siRNA over the whole recording period, from t = 0 s to t = 60 s as indicated; the corresponding calcium signal-traces are shown in (**d**); scale bar = 10 µm. (**f**) Representative traces of normalized fluorescence intensities (F.I.) of a neuron from the culture transfected with *DNMT1* siRNA. Left trace shows an active cell (solid calcium oscillation). Right trace shows an inactive cell (no observable calcium oscillations). (**g**) Bulk quantification of the percentage of active and inactive neurons of control and *DNMT1* siRNA transfected cultures (Mean ± SEM). Up to 39 acquisition ROIs (with >3280 cells) were analyzed for every condition in four independent experiments. A two-tailed unpaired *t*-test comparing the percentage of active cells in control vs. *DNMT1* siRNA transfected neurons was performed as statistical analysis; *** = *p* ≤ 0.001.

Having successfully differentiated the human iPSCs into spontaneously active glutamatergic cortical neurons, we next analyzed the *DNMT1* siRNA efficacy to downregulate *DNMT1* expression in human cells. We transfected HEK293T cells with either control siRNA or a mixture of control and *DNMT1* siRNA by the use of lipofection. In accordance with our transfection protocol for iPSC-derived cortical neurons, expression levels of *DNMT1* were assessed 24 h after transfection. For this, RNA was isolated from transfected cells and transcribed into cDNA by reverse transcription. Subsequently, this cDNA was measured in a quantitative real-time PCR using *DNMT1*-specific primers ([29], see methods for primer sequences). Utilizing the ΔΔCt-method, we normalized the mean Ct-values of conditions with *DNMT1* primers to the respective conditions using *RPS18*-primers, encoding for the ribosomal 40S subunit, which is routinely used as house-keeping gene [30]. Compared to cells treated only with control siRNA, the level of *DNMT1* transcripts in cells, transfected with *DNMT1* siRNA, was significantly reduced, confirming a successful knockdown (Figure 2a). We previously showed that such a siRNA-mediated depletion not only results in reduced transcript levels, but similarly diminishes DNMT1 protein levels within 24 h [31,32].

### 2.2. DNMT1 Depletion Reduces Spontaneous Neuronal Activity in Human iPSC-Derived Cortical Excitatory Neurons

After validation of knockdown efficiency of *DNMT1* siRNA in HEK cells, we aimed to reduce *DNMT1* levels in our neuronal cultures to assess the impact of DNA methylation mediated by DNMT1 on the synaptic activity of human excitatory neurons. Therefore, two weeks old neuronal cultures were transfected with target-specific *DNMT1* siRNA oligos or control siRNA, and spontaneous calcium oscillations were analyzed by live-cell imaging (Figure 2b–g). Calcium imaging relies on the visualization of intracellular calcium, which in our experimental setup reflects mainly cytosolic calcium due to the used calcium-sensitive dye Fluo-4 and the short loading time (20 min). Observed changes in the cytosolic calcium levels reflect the intensity and frequency of neuronal activity. As we (i) do not provide any external cues or stimuli inducing membrane depolarization, and (ii) are using highly homogenous cultures of glutamatergic neurons (see Figure 1 and [25]), the observed calcium oscillations, indicative of spontaneous neuronal activity, are mainly due to glutamatergic excitation.

Calcium levels of four independent cultures of iPSC-derived glutamatergic excitatory neurons (500–1300 cells per culture) were recorded for 1 min and analyzed manually (see Supplementary Files A2 and A3). Neurons were categorized according to the presence (= active cell) or absence (= inactive cell) of visual calcium oscillations (Figure 2b,c, Supplementary Files A1 and A2). Active cells are characterized by high calcium levels, which is indicated by red color in pseudo-colored images (Figure 2b,c). Persistent low levels of calcium and absence of calcium oscillations seen in inactive cells are visualized by blue to green colors in pseudo-colored images (Figure 2b,c). To confirm the visual categorization of cells into active and inactive states, normalized fluorescence intensity (F.I.) changes of single cells were plotted over 60 s (Figure 2d–f). Active neurons show a recurrent change in F.I. (=oscillations), while inactive cells have a relatively constant and low F.I. without recognizable spikes, which is also confirmed by pseudo-colored time-series images of these cells (Figure 2d–f). We analyzed the percentage of active and inactive cells as a fraction of all recorded cells, based on our categorization (Figure 2g). In the absence of external stimuli, 49.4 ± 1.5% of neurons transfected with control siRNA showed spontaneous activity indicated by F.I. changes of Fluo-4. Interestingly, *DNMT1* depletion by RNAi resulted in a significantly lower proportion of spontaneously active neurons (31.3 ± 2.8%) already 24 h after transfection. Thus, reminiscent of what was reported in murine neurons, DNMT1 modulates the synaptic activity of human cortical neurons.

However, given by the fact that the oscillations in the F.I. investigated here integrate the whole network activity, of which not all cells are transfected equally, further studies are required to nail down the exact mechanisms and to dissect whether DNMT1 similarly acts on synaptic vesicle release as well as the subsequent replenishment.

## 3. Discussion

The here presented data, acquired in excitatory neurons differentiated from human iPSCs, propose a positive correlation of DNMT1-levels and associated net-activity. Such a positive correlation of DNMT1 expression and neuronal network activity in excitatory neurons resembles a direct reflection of previous findings in mice. There, in murine hippocampal cell cultures, the inhibition of DNMT activity resulted in a reduced frequency of miniature excitatory postsynaptic currents [17].

In contrast to this, previous work from our group indicated a reciprocal conjunction of synaptic activity and DNA methylation or DNMT activity in murine inhibitory cortical interneurons. *Dnmt1* deletion in these cells resulted in increased frequencies of mIPSCs and upregulated GABAergic transmission [20]. Hence, DNMT1 could mediate opposing synaptic functions in excitatory cortical neurons versus inhibitory interneurons. However, this would need to be determined in detail electrophysiologically in future studies. While DNMT1 seems to restrict the activity of GABAergic interneurons, neuronal activity of excitatory neurons appears to be promoted. Thus, in both cases DNMT1 would lead to enhanced excitation on the network level. Interestingly, the expression levels of DNMT1 and DNMT3a were shown to be significantly increased in TLE patients [23]. Such an elevated expression could lead to a net-increase in neuronal firing by diminished inhibition and increased excitation, assuming DNMT1 has similar functions in human inhibitory interneurons as described for mice. Modifications of a neuronal system’s net-excitation can be associated with pronounced pathologies, such as epilepsy, neuronal cell death, and neurodegeneration in case of aberrant excitation, or loss of cognitive function in case of aberrant inhibition. Hence, by driving excitation DNMTs and DNA methylation could be implicated in promoting synaptic plasticity and memory formation, and also drive epileptic seizures and convulsions when being overexpressed. However, to draw final conclusions, verification of DNMT function in human inhibitory interneurons needs to be conducted, for which likewise human iPSC-derived interneurons could be used. Moreover, it would be interesting to dissect the detailed mechanisms through which DNMTs regulate synaptic function in human excitatory versus inhibitory cortical neurons. Apart from DNA methylation, we and others showed that DNMT1 acts noncanonically, interfering with histone modifying complexes [15,31,33]. Deciphering the mode of action and the target genes would help to understand disease pathophysiology and develop targeted therapy strategies.

It further needs to be considered that not only DNA methylation regulates synaptic function, but vice versa that altered neuronal activity patterns also modify DNA methylation signatures [4]. This could result from the activated execution of processes involved in the degradation of DNA-methylating proteins, as already described for DNMT3A1 [22]. Different DNMTs and isoforms have been reported to display different binding specificities. By structural, functional, and cellular characterization of DNMT3A and DNMT3B, a multilayered substrate-recognition mechanism was identified that accounts for their divergent genomic methylation activities [34,35,36]. Thus, the temporary change in the availability of particular DNMTs potentially contributes to the dynamic modulation of DNA methylation changes of specific genes, e.g., being associated with synaptic plasticity.

In addition to this, the activity and targeting of DNMTs to discrete genomic loci could be distinctively affected by altered neuronal activity. Herein, lncRNA-mediated sequestration of DNMTs or prevention of DNMT binding represent possible mechanisms through which the targeting of DNMTs might be regulated [37,38]. Antisense transcription, complementary base pairing, or triplex-structure formation localize lncRNAs to discrete genomic loci [38]. As lncRNA expression has been reported to be responsive to altered neuronal activity, and as they are known to interact with DNMTs and other chromatin remodelers [5,6,39], they emerge as powerful mediators of recruiting DNMTs to particular genomic loci upon changed activity levels.

In conclusion, epigenetic mechanisms facilitate a high level of versatility and adaptability, enabling neurons to adapt to and integrate changing activity patterns. Such mechanisms of transcriptional control own an extensive reach regarding their potential to dynamically influence neuronal function. Their mode of action is especially of interest to explain cellular processes, such as neuronal information processing that require a high level of flexibility, but also persistence. With this study, we demonstrate that interfering with the expression of DNMT1 is already sufficient to reduce the neuronal net-activity of human iPSC-derived cortical excitatory neurons. Hence, precise fine-tuning of DNMT1 function and DNA methylation in various neuronal subtypes might be a prerequisite for proper neuronal function, learning, and long-term memory. Even subtle impairments will likely have detrimental consequences for cognitive function, supporting the important role already described for DNA methylation and DNMTs in neuropsychiatric diseases [40].

## 4. Materials and Methods

### 4.1. Isolation and Cultivation of Primary Murine Astrocytes

For the isolation of primary murine astrocytes, brains of FVB/N mouse embryos were dissected at embryonic day 13.5. Brainstem and meninges were removed and whole cortex was digested with 1× Trypsin (PAN-Biotech, Aidenbach, Germany) for 7 min at 37 °C. Astrocytes were diluted in prewarmed (37 °C) glial plating medium (Neurobasal media (Thermofisher Scientific, Waltham, MA, USA), 10% FBS, 1× Antibiotic-/Antimycotic solution (Thermofisher Scientific), seeded into Poly-D-Lysine (Sigma Aldrich, St. Luis, MO, USA) coated T75 flasks and cultivated in a humidified incubator at 37 °C, 5% CO_2_. Medium was changed once a week and astrocytes were passaged routinely. For the co-cultivation of astrocytes with hiPSC-derived neurons, glia cells were trypsinized for 5 min at 37 °C and harvested by centrifugation at 400× *g* for 5 min. After counting with trypan blue, cells were added to the human cortical neuron cultures.

### 4.2. iPSC Maintenance and Differentiation into Cortical Neurons

WTC11 cells with a doxycycline-inducible *Ngn2* transgene [24,25] were cultured in StemMacs iPS-Brew XF Medium (Miltenyi Biotec, Bergisch Gladbach, Germany) on GelTrex-coated plates (Thermofisher Scientific) with medium changes every other day. Cells were passaged regularly, using Versene (Thermofisher Scientific) and Thiazovivin-supplemented medium (Axon Medchem, Groningen, Netherlands) for the first 24 h. Differentiation into cortical neurons was performed as described before with slight modifications [25]. In brief, 1.5 × 10^6^ cells were seeded onto GelTrex coated 6-well plates in predifferentiation medium (KO-DMEM/F12 (Thermofisher Scientific), 1× N2 supplement (PAN-Biotech), 1× non-essential amino acids (NEAA, Thermofisher Scientific), 10 ng/mL BDNF (Peprotech, London, UK), 10 ng/mL NT3 (Peprotech), 1 µg/mL Laminin (Sigma-Aldrich), 2 µg/mL doxycycline (Sigma-Aldrich), and 1x antibiotic/antimycotic solution (Sigma-Aldrich)) supplemented with Thiazovivin (day prior to differentiation −3). After 24 h (day −2) and 48 h (day −1), medium was changed to fresh predifferentiation medium without Thiazovivin. On day 0, 2.5 × 10^5^ cells were seeded onto GelTrex-coated coverslips (in 6-well plates) in maturation medium (50% KO-DMEM/F12, 50% Neurobasal medium (Thermofisher Scientific), 0.5× N2 supplement, 1× NEAA, 0.5× GlutaMax supplement (Thermofisher Scientific), 0.5× B27 supplement (Thermofisher Scientific), 10 ng/mL BDNF, 10 ng/mL NT3, 1 µg/mL Laminin, 2 µg/mL doxycycline, and 1× antibiotic/antimycotic solution) supplemented with 1:100 GelTrex. On day 2, mouse primary astrocytes were added to the neurons in a neuron-to-glia ratio of 2:1. Cultures were treated with 2 µM Cytosine β-D-arabinofuranoside (Ara-C, Sigma-Aldrich) on day 4. From day 6 on, 50% of media was changed every other day to fresh maintenance media. From day 9 onwards, half of media was removed every other day and exchanged with maintenance medium supplemented with 2.5% fetal bovine serum.

### 4.3. Immunocytochemistry of iPSC-Derived Neuronal Cultures

To characterize iPSC-derived neuronal cultures, neurons were fixed with 3.7% FA in 4% sucrose 14 days after the start of differentiation (d14). After blocking and permeabilization, neurons were stained with primary and secondary antibodies as described before [41]. The following antibodies were used: anti-TAU (rabbit, K9JA, Dako (Jena, Germany), A0024), anti-MAP2 (chicken, Abcam (Camebridge,. UK), ab5392), anti-vGlut1 (mouse, Merck Millipore (Burlington, MA, USA), MAB5502), anti-ChAT (rabbit, Thermofisher Scientific, PA5-26597), anti-SYP (mouse, Santa Cruz Biotechnology (Dallas, TX, USA), sc-17750), anti-vGLUT1 (rabbit, Synaptic Systems (Göttingen, Germany), 135303), anti-PSD95 (mouse, Biolegend (San Diego, CA, USA), MMS-5182), anti-rabbit IgG Alexa-Fluor-488 (donkey, Thermofisher Scientific, A21206), anti-mouse IgG Alexa-Fluor-488 (donkey, Thermofisher Scientific, A21202), anti-chicken IgG AlexaFluor-647 (goat, Thermofisher Scientific, A21449), anti-rabbit IgG AlexaFluor-568 (goat, Thermofisher Scientific, A11011), anti-mouse IgG AlexaFluor-568 (donkey, Thermofisher Scientific, A10037).

### 4.4. siRNA Oligo Transfection

iPSC-derived neurons were transfected with LipoStem (Thermofisher Scientific) on day 13 or 14 of differentiation according to the manufacturer’s protocol. Half of the culture medium was removed and stored before transfection. Transfection of cells was conducted using 15 nM control siRNA (BLOCK-iT Alexa Fluor red fluorescent oligo, 14750100; Invitrogen (Carlsbad, CA, USA)) or 30 nM *DNMT1* siRNA (sc-35204; Santa Cruz Biotechnology). Then, 24 h after transfection, the medium was changed to previously collected conditioned medium, and cells were transferred to a live-imaging chamber (ALA Scientific Instruments, Farmingdale, NY, USA) to perform live-cell calcium imaging. To roughly estimate the transfection efficiency, cotransfection of cells was conducted using 15 nM control siRNA and 30 nM *DNMT1* siRNA as above, and transfected cells were manually counted based on the presence of red fluorescence, yielding a transfection efficiency of 39.1 ± 2.3% 24 h after transfection.

### 4.5. Live-Cell Calcium Imaging

To analyze spontaneous Ca^2+^ oscillations, iPSC-derived neurons were labeled with 2 µM Fluo-4 (Thermofisher Scientific) and 0.2% Pluronic F127 (Merck Millipore, Darmstadt, Germany) for 20 min, 24 h after siRNA transfection. Time-lapse movies of up to 10 different fields (=acquisition ROI) were recorded for 1 min each (framerate: 1 Hz). Cells of all acquisition ROIs were counted and subdivided into active and inactive cells by visual inspection of the presence (=active) or absence (=inactive) of fluorescence intensity changes over time. The percentage of active cells was calculated as the fraction of active cells from the total cell count in an acquisition ROI. Up to 39 acquisition ROIs (with >3280 cells) were analyzed for every condition in four independent experiments. A two-tailed unpaired *t*-test comparing the percentage of active cells with control or *DNMT1* siRNA transfection was performed for statistical analysis. Statistical analysis was performed using GraphPad Prism v8.0.1 software (San Diego, CA, USA).

### 4.6. Cultivation Conditions and Treatment of HEK Cells

Human embryonic kidney 293T (HEK) cells were cultured in high-glucose DMEM (Invitrogen) supplemented with 10% FBS at 37 °C, 95% humidity and 5% CO_2_. Prior to any treatment, cells were seeded into 6-well plates at a density of 8 × 10^4^ cells per well.

The siRNA transfection was performed 24 h after seeding, using Lipofectamine 2000 (Thermo Fisher Scientific) according to the manufacturer’s protocol for reverse lipofection and 15 nM control siRNA (BLOCK-iT Alexa Fluor red fluorescent oligo, 14750100; Invitrogen) or 30 nM *DNMT1* siRNA (sc-35204; Santa Cruz Biotechnology) for 5 h in Opti-MEM I reduced serum medium (Thermo Fisher Scientific). Subsequently, cells were again cultured in the previously described medium for 24 h at 37 °C, 95% humidity and 5% CO_2_.

### 4.7. RNA Isolation and Quantitative Reverse Transcription PCR (qRT-PCR)

For RNA expression analysis, siRNA-treated HEK cells were harvested using TRIzol Reagent (Life Technologies) according to the manufacturer’s protocol, frozen, and stored at −20 °C. Isolated RNA quantities were evaluated using a Qubit 4 Fluorometer (Thermo Fisher Scientific) and were used for cDNA synthesis utilizing the iScript cDNA Synthesis Kit (BioRad). Quantitative PCR reactions for analyzing DNMT1 quantities were performed using 10 ng cDNA per sample and the PowerUp SYBR Green Mastermix (Thermo Fisher Scientific) on the CFX96 thermocycler (BioRad). Sequences of the utilized primers were as follows (indicated as 5′ to 3′, forward = fw, reverse = rev): DNMT1 fw CCATCAGGCATTCTACCA, DNMT1 rev CGTTCTCCTTGTCTTCTCT, RPS18 fw GTTCCAGCATATTTTGCGAGT, RPS18 rev GTCAATGTCTGCTTTCCTCAAC. Data were normalized to RPS18 and analyzed via the ΔΔCt-method.

## Data Availability

The data presented in this study are available on request from the corresponding authors. The data are not publicly available due to very large size for either high resolution microscopy and time lapse movies.

## Supplementary Materials

Supplementary materials can be found at https://www.mdpi.com/1422-0067/22/4/2034/s1.
